# Efficacy of Two Rotary NiTi Instruments in Removal of Resilon/Epiphany Obturants

**Published:** 2012-10-13

**Authors:** Bahareh Dadresanfar, Mahbubeh Iranmanesh, Pooneh Mohebbi, Payman Mehrvarzfar, Mehdi Vatanpour

**Affiliations:** 1. Department of Endodontics, Dental Branch, Islamic Azad University of Medical Sciences, Tehran, Iran.; 2. Private Practice, Tehran, Iran.

**Keywords:** Retreatment, Resilon Sealer, Mtwo R, ProTaper D series

## Abstract

**Introduction:**

The success of endodontic retreatment is related to the complete removal of the obturation material from the root canal system. The aim of this study was to evaluate the efficacy of Mtwo R and ProTaper retreatment files in removing the Resilon/Epiphany system with or without chloroform during retreatment.

**Materials and Methods:**

Sixty distal roots of first mandibular molars were prepared and laterally condensed with Resilon/Epiphany, then divided into four groups (15 each for retreatment): 1) Mtwo R/solvent; 2) Mtwo R; 3) ProTaper D/solvent; and 4) ProTaper D. The cleanliness of the canal walls was evaluated using radiography; a stereomicroscope and SEM. Data were subjected to ANOVA and Student’s t-test.

**Results:**

Neither rotary system performed better than the other when considering the whole root canal, with or without solvent. In the apical portion, ProTaper/solvent showed the best result (P<0.05).

**Conclusion:**

In Resilon/Epiphany retreatment cases, ProTaper/solvent was better in the apical portion; however when considering the whole canal, Mtwo R and the ProTaper D series had the same performance.

## Introduction

Endodontic retreatment is considered successful only if the obturation material is removed thoroughly from the root canal system and replaced with an appropriate filling material [1][2][3][4].

Several materials have been used to fill root canals, with gutta-percha being the most popular. However, gutta-percha has two major drawbacks: poor sealing ability and inability to further strengthen the teeth [5]. To overcome these shortcomings, in recent years a thermoplastic synthetic polymer-based root canal filling material, Resilon (Pentron Clinical Technologies, Wallingford, CT, USA), has been developed. Resilon includes bioactive glass and radiopaque fillers. It performs like gutta-percha, has the same handling properties and can be softened with heat or solvents like chloroform for re-treatment purposes. Resilon points and Epiphany sealer, (Pentron Clinical Technologies, Wallingford, CT, USA), adhere to one another and to the root canal walls, thus forming a “monoblock” structure [6][7].

To date, different methods have been used to remove root canal filling materials including endodontic hand instruments, heat, solvents, Gates-Glidden burs, ultrasonic instruments, nickel-titanium (NiTi) rotary instruments and lasers [8][9][10][11].

Several studies have evaluated the efficacies of different NiTi rotary systems in the removal of root canal filling materials, whereby these systems promised reduced working time [9][12]. Removal of Epiphany/Resilon with NiTi rotary files has also been investigated [4][13][14], although the efficacy of this method has not yet been fully established.

Two NiTi rotary file systems have recently been introduced and are specifically designed for removing semisolid filling materials: Mtwo R (retreatment) rotary files (Sweden and Martina, Padova, Italy) and ProTaper Universal retreatment files (Dentsply-Maillefer, Ballaigues, Switzerland). These two systems have not yet been compared during retreatment of the Resilon/Epiphany system in the absence or presence of chloroform.

It has been shown that chloroform usage reduces the time of retreatment and also the amount of residue [15]. The aim of this study was to evaluate the efficacy of these two NiTi rotary instruments, Mtwo R and ProTaper Universal retreatment files, with or without Chloroform in removing Resilon/Epiphany as the root filling material.

## Materials and Methods

Sixty human extracted mandibular first molars were decoronated at the CEJ after washing and storing in 0.1% thymol. Average root length was 16 mm with curvature less than 20° [16]. Only the distal canal root was instrumented, obturated and retreated.

### Canal preparation and obturation

Working length (WL) was determined by introducing a #10 K-file (Dentsply Maillefer, Ballaigues, Switzerland) into the canal until it could be seen at the apical foramen and subtracting 1 mm from the acquired length. The samples were prepared using the step back technique with sequential use of K-files. Circumferential hand filing was conducted up to a #35 file at WL and flaring was carried out by decreasing 0.5 mm from the last file until a #60 file was reached. During instrumentation, the canals were irrigated with 30 mL of 5.25% NaOCl. The smear layer was removed by irrigating with 17% EDTA followed by 5.25% NaOCl, and the canals were finally rinsed with 10 ml of distilled water. After drying the canals with #35 paper points, obturations were done laterally with #35 Resilon cones (Pentron Clinical Technologies, Wallingford, CT, USA) as master apical cones, #15 cones as accessories and Epiphany sealer (Pentron Clinical Technologies, Wallingford, CT, USA) according to the manufacturer. The coronal portion was light cured and temporarily sealed with Coltosol (Coltene, Altstatten, Switzerland). Two radiographs were taken mesiodistally and buccolingually (15 cm distance, 0.4 s). The roots were then incubated at 37°C for three weeks.

The coronal 2-3 mm of the filling material was removed with a #2 Gates-Glidden bur (Dentsply, Maillefer, Ballaigues, Switzerland). Then the samples were randomly divided into four groups with 15 teeth in each group. An electric motor (Endo IT motor; VDW, Munich, Germany) was used for each rotary file according to the manufacturer’s instructions.

### Group A: Mtwo R/solvent

Two drops of chloroform (Kimia Co. Tehran, Iran) from a tuberculin syringe were applied before insertion of each instrument and were re-applied during instrumentation, if necessary. Mtwo R size 05/25 followed by 05/15 were penetrated into the canal until no Resilon could be extruded. Final preparation was done with a Mtwo R size 04/35 file followed by 04/40 file. Between using each instrument, the canals were irrigated using 5.25% NaOCl.

### Group B: Mtwo R

The retreatment procedure was conducted identical to group A except chloroform was not applied.

### Group C: ProTaper/solvent

Two drops of chloroform were applied before insertion of each instrument and were re-applied during instrumentation if necessary. ProTaper Universal retreatment files (D1, D2, D3) were used in a crown-down technique. Size D3 was used to the working length until no Resilon was extruded from the canal, and final preparation was performed using ProTaper size F4 (05/40).

### Group D: ProTaper

The retreatment procedure was same as group C except chloroform was not applied.

In order to assess complete removal of obturation materials, two radiographs were taken mesiodistally and buccolingualy. If the treatment procedure was radiographically deemed to be incomplete, we repeated the instrumentation with the last file used in each group until no residual filling material could be detected. Roots were split longitudinally in two halves by chisel, taking care not to enter the canal space. The amount of remaining Resilon/Epiphany was evaluated in three segments: 1 mm from the apex (apical), 8 mm from the apex (middle) and 2 mm below the CEJ (coronal) using a stereomicroscope (Olympus, SZM9, NY, USA) at 16× magnification. In each section the remaining filling material was measured as a percentage of the dentinal wall of each third using AutoCAD 2007 (Autodesk Inc., San Rafael, CA, USA). Sections showing no remaining material were prepared to be evaluated under a scanning electron microscope (SEM) (Leo. 440i; Oxford Microscopy, Oxford, UK) using a scattered electron (SE) detector at 30x magnification.

Two way ANOVA, repeated measure ANOVA, t-test, χ² and Fisher’s exact test were used to analyze the data. The significance level was set at P=0.05.

## Results

When analyzing the whole canal there was no significant difference between groups with or without the solvent.

Although the coronal and middle thirds did not show any significant differences between groups (P>0.005), the apical third in the ProTaper/solvent group showed the least residue in comparison to the same third in the other groups (P=0.005).

**Figure 1:**
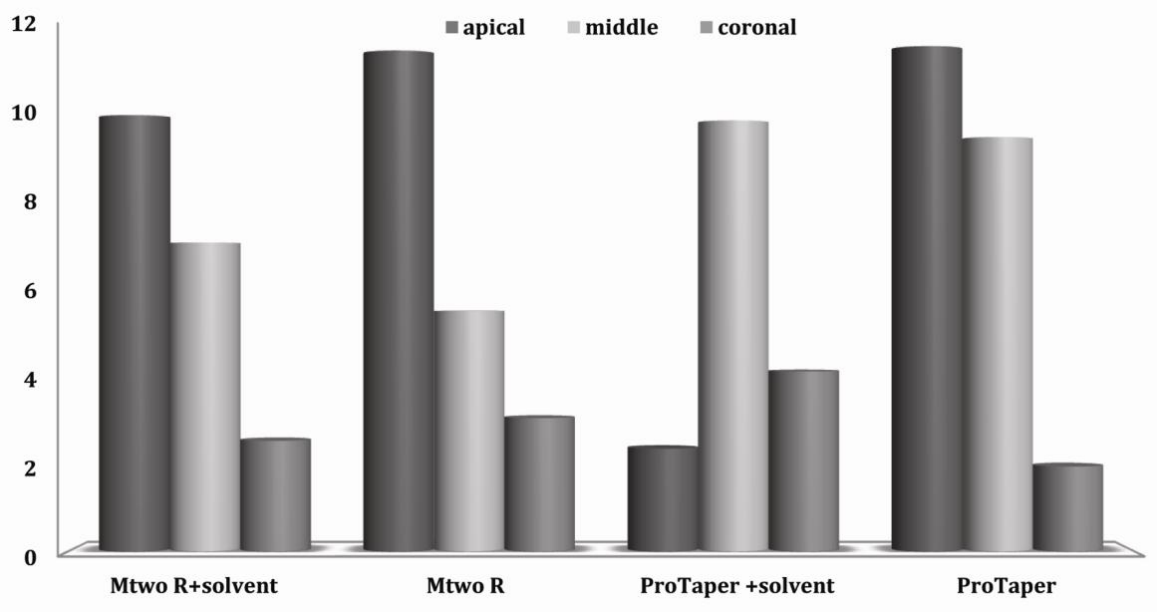
Mean value and standard error of percentage of debris in each third

Comparisons of these four groups according to the mean value ± standard error of each root third are presented in Figure 1.

Considering each group individually, in the ProTaper/solvent group, there was significantly more remnant filling material in the middle third than in the apical (P=0.003) or coronal thirds (P=0.027). Furthermore, with the Mtwo R, Mtwo R+solvent and ProTaper groups, significantly more filling material remained in the apical third than in the coronal third (P<0.05).

Table 1 shows the samples analyzed by SEM.

**Table 1:** Samples analyzed by SEM according to the residue presence

**Groups**	**N **a	**+** b	**- **c
**Mtwo R/solvent**	13	3	10
**Mtwo R**	10	2	8
**ProTaper/solvent**	9	1	8
**ProTaper **	10	4	6

^a^ N:total number

^b^ +:filling residue detected

^c^ -:filling residue not detected

**Figure 2:**
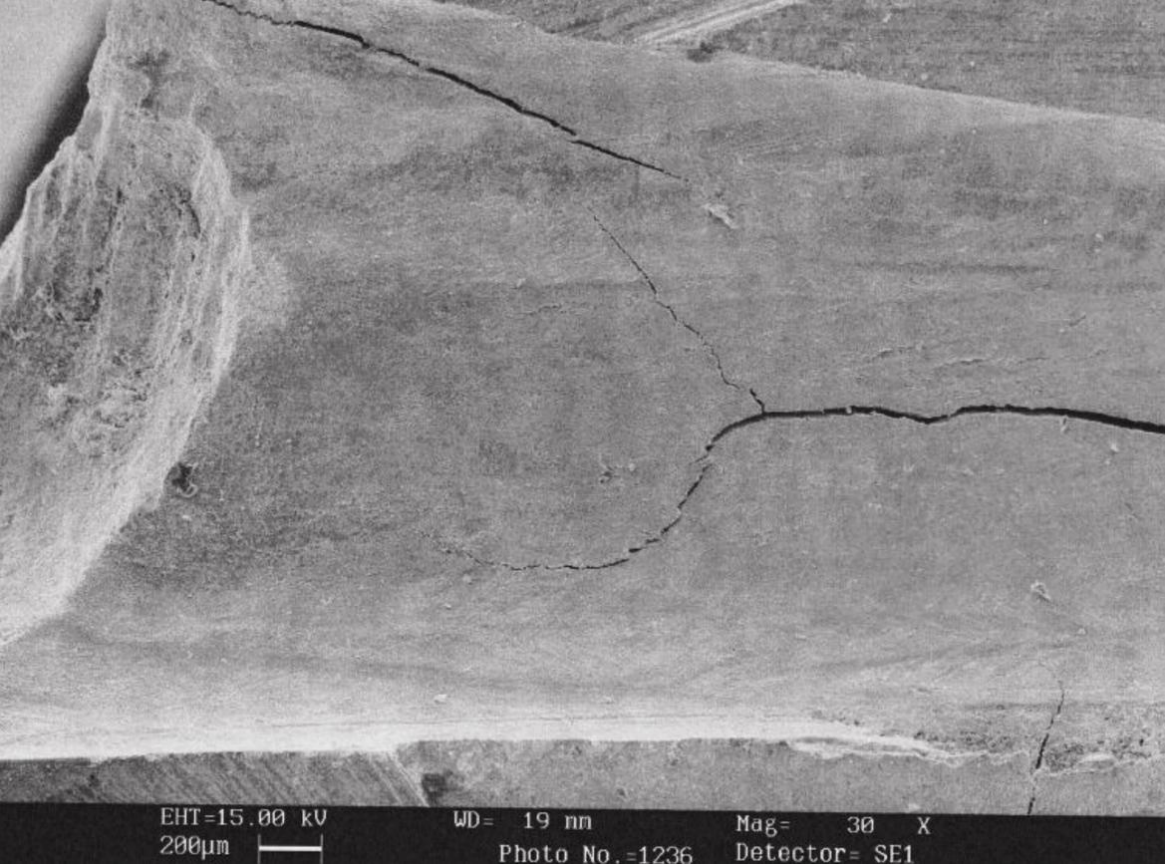
SEM micrograph of the middle portion of a tooth in ProTaper D group

Following evaluation with SEM (Figure 2), there was no significant difference among groups in detecting Resilon (P=0.64). When comparing stereomicroscopy and SEM in detecting Resilon remnant, SEM performed better than the stereomicroscope (P<0.001).

## Discussion

Thorough removal of the root filling material during the retreatment procedure is necessary to eliminate as much necrotic material and bacterial remnants as possible from the root canal space [17]. Thorough removal of the root filling material is difficult and essential this concern is greatest in the apical portion, where the most infectious residue is found.

In this study, ProTaper D and Mtwo R files, two newly introduced rotary files designed for retreatment purposes, were used. Due to their cutting tips, they penetrate more easily into the filling mass and reduce mishaps such as file separation through penetration [18].

Retreatment of canals obturated with resin based materials such as Resilon/Epiphany may be a challenge due to their bond strength to the root surface and their penetration into lateral canals and dentinal tubules [19][20]. Although the manufacturer recommends Chloroform application in order to enhance the retreatment efficacy of the epiphany system, we compared retreatment of this system with and without Chloroform. This was due to the disadvantages attributed to Chloroform such as local toxicity in contact with periradicular tissues [21] and adverse effects on the bond strength of resin based material after root canal re-obturation [19].

According to our study, the retreatment of Resilon/epiphany was found to be possible and efficient even without the use of the solvent throughout the canal; this finding was in accordance with the study performed by Schirrmeister et al. [4]. To help remove as much filling material as possible, it is necessary to enlarge the canal to a size larger than the pre-obturation size [15][17]; so we used #40 for both Mtwo and ProTaper as the final file, which prepared the canals one size larger than the initial size (#35). Therefore the similarity shown in the present study between groups with and without solvent can be attributed to the enlargement of the root canals during the retreatment procedure which can remove resin tags, rather than the effect of Chloroform.

We found the least residue in the apical portion of the ProTaper/solvent group. The reason may be due to the difference in taper in the final file used between the ProTaper (0.05) and Mtwo groups (0.04). This may have allowed more solvent penetration into the apical portions. In comparative studies, ProTaper D files proved to be more effective in removing obturation material from the root canal compared to Mtwo R [22][23].

There are several methods for assessing root filling residue after retreatment, including radiography, photography, SEM, computed tomography, clearing roots, dissolution and using microscopes [24][25][26][27][28][29]. In the present study, we confirmed retreatment completion by taking radiographs to simulate the clinical condition; then we checked the samples under stereomicroscope and SEM to minimize the subjectivity of radiographs, as in Cunha et al.’s [25] and Horvath et al. study [30]. Under SEM, obturation material remnants were detected in all three parts, despite their absence in the radiographs and stereomicroscope images. This was in accordance with the comparative studies [25][30].

None of the groups exhibited complete removal of the filling material, consistent with previous studies [4][13][14][15][25][26][27][31]. Although detecting obturation remnants with SEM may seem to have no clinical relevance, this can indicate the inefficacy of our available files in completely removing filling material during retreatment, which is in accordance with other studies [15][31][32].

## Conclusions

We recommend limiting chloroform application to the apical third, especially while using ProTaper. Under the conditions of this in vitro study, the Mtwo R and ProTaper D series were similar in removing Resilon/Epiphany filling material during retreatment, with or without chloroform, when considering the whole canal.

## References

[1] Bergenholtz G, Lekholm U, Milthon R, Heden G, Odesjo B, Engstrom B (1979). Retreatment of endodontic fillings. Scand J Dent Res.

[2] Friedman S, Stabholz A, Tamse A (1990). Endodontic retreatment--case selection and technique. 3. Retreatment techniques. J Endod.

[3] Stabholz A, Friedman S (1988). Endodontic retreatment--case selection and technique. Part 2: Treatment planning for retreatment. J Endod.

[4] Schirrmeister JF, Meyer KM, Hermanns P, Altenburger MJ, Wrbas KT (2006). Effectiveness of hand and rotary instrumentation for removing a new synthetic polymer-based root canal obturation material (Epiphany) during retreatment. Int Endod J.

[5] Goodman A, Schilder H, Aldrich W (1974). The thermomechanical properties of gutta-percha. II. The history and molecular chemistry of gutta-percha. Oral Surg Oral Med Oral Pathol.

[6] Mohammadi Z, Khademi A (2007). An evaluation of the sealing ability of MTA and Resilon: A bacterial leakage study. Iran Endod J.

[7] Teixeira FB, Teixeira EC, Thompson JY, Trope M (2004). Fracture resistance of roots endodontically treated with a new resin filling material. J Am Dent Assoc.

[8] Hammad M, Qualtrough A, Silikas N (2008). Three-dimensional evaluation of effectiveness of hand and rotary instrumentation for retreatment of canals filled with different materials. J Endod.

[9] Masiero AV, Barletta FB (2005). Effectiveness of different techniques for removing gutta-percha during retreatment. Int Endod J.

[10] Viducic D, Jukic S, Karlovic Z, Bozic Z, Miletic I, Anic I (2003). Removal of gutta-percha from root canals using an Nd:YAG laser. Int Endod J.

[11] Wilcox LR (1989). Endodontic retreatment: ultrasonics and chloroform as the final step in reinstrumentation. J Endod.

[12] Hulsmann M, Bluhm V (2004). Efficacy, cleaning ability and safety of different rotary NiTi instruments in root canal retreatment. Int Endod J.

[13] de Oliveira DP, Barbizam JV, Trope M, Teixeira FB (2006). Comparison between gutta-percha and resilon removal using two different techniques in endodontic retreatment. J Endod.

[14] Ezzie E, Fleury A, Solomon E, Spears R, He J (2006). Efficacy of retreatment techniques for a resin-based root canal obturation material. J Endod.

[15] Hassanloo A, Watson P, Finer Y, Friedman S (2007). Retreatment efficacy of the Epiphany soft resin obturation system. Int Endod J.

[16] Schneider SW (1971). A comparison of canal preparations in straight and curved root canals. Oral Surg Oral Med Oral Pathol.

[17] Friedman S, Stabholz A (1986). Endodontic retreatment--case selection and technique. Part 1: Criteria for case selection. J Endod.

[18] Inan U, Aydin C (2012). Comparison of cyclic fatigue resistance of three different rotary nickel-titanium instruments designed for retreatment. J Endod.

[19] Shokouhinejad N, Sabeti MA, Hasheminasab M, Shafiei F, Shamshiri AR (2010). Push-out bond strength of Resilon/Epiphany self-etch to intraradicular dentin after retreatment: a preliminary study. J Endod.

[20] Shokouhinejad N, Sabeti M, Gorjestani H, Saghiri MA, Lotfi M, Hoseini A (2011). Penetration of Epiphany, Epiphany self-etch, and AH Plus into dentinal tubules: a scanning electron microscopy study. J Endod.

[21] Barbosa SV, Burkard DH, Spangberg LS (1994). Cytotoxic effects of gutta-percha solvents. J Endod.

[22] Marques da SB, Baratto-Filho F, Leonardi DP, Henrique BA, Volpato L, Branco BF (2012). Effectiveness of ProTaper, D-RaCe, and Mtwo retreatment files with and without supplementary instruments in the removal of root canal filling material. Int Endod J.

[23] Yilmaz Z, Karapinar SP, Ozcelik B (2011). Efficacy of rotary Ni-Ti retreatment systems in root canals filled with a new warm vertical compaction technique. Dent Mater J.

[24] Azar M, Khojastehpour L, Iranpour N (2011). A comparison of the effectiveness of chloroform in dissolving resilon and gutta-percha. J Dent (Tehran )..

[25] Cunha RS, de Martin AS, Barros PP, da Silva FM, Jacinto RC, Bueno CE (2007). In vitro evaluation of the cleansing working time and analysis of the amount of gutta-percha or Resilon remnants in the root canal walls after instrumentation for endodontic retreatment. J Endod.

[26] Shokouhinejad N, Ramzi H, Saghiri MA, Samieefard A (2010). Efficacy of three different methods in retreatment of root canals filled with Resilon/Epiphany SE. Iran Endod J.

[27] Marfisi K, Mercade M, Plotino G, Duran-Sindreu F, Bueno R, Roig M (2010). Efficacy of three different rotary files to remove gutta-percha and Resilon from root canals. Int Endod J.

[28] Tasdemir T, Yildirim T, Celik D (2008). Comparative study of removal of current endodontic fillings. J Endod.

[29] Akhavan H, Azdadi YK, Azimi S, Dadresanfar B, Ahmadi A (2012). Comparing the Efficacy of Mtwo and D-RaCe Retreatment Systems in Removing Residual Gutta-Percha and Sealer in the Root Canal. Iran Endod J.

[30] Horvath SD, Altenburger MJ, Naumann M, Wolkewitz M, Schirrmeister JF (2009). Cleanliness of dentinal tubules following gutta-percha removal with and without solvents: a scanning electron microscopic study. Int Endod J.

[31] Fenoul G, Meless GD, Perez F (2010). The efficacy of R-Endo rotary NiTi and stainless-steel hand instruments to remove gutta-percha and Resilon. Int Endod J.

[32] Dadresanfar B, Mehrvarzfar P, Saghiri MA, Ghafari S, Khalilakn Z, Vatanpour M (2011). Efficacy of two rotary systems in removing guttapercha and sealer from the root canal walls. Iran Endod J.

